# Prognosis-Predictive Signature and Nomogram Based on Autophagy-Related Long Non-coding RNAs for Hepatocellular Carcinoma

**DOI:** 10.3389/fgene.2020.608668

**Published:** 2020-12-23

**Authors:** Yu Jia, Yan Chen, Jiansheng Liu

**Affiliations:** ^1^Department of General Surgery, First Hospital of Shanxi Medical University, Taiyuan, China; ^2^First Clinical Medical College, Shanxi Medical University, Taiyuan, China

**Keywords:** hepatocellular carcinoma, autophagy, long non-coding RNAs, The Cancer Genome Atlas, prognostic signature, nomogram

## Abstract

Autophagy plays a vital role in hepatocellular carcinoma (HCC) pathogenesis. Long non-coding RNAs (lncRNAs) are considered regulators of autophagy, and the aim of the present study was to investigate the prognostic value of autophagy-related lncRNA (ARlncRNA) and develop a new prognostic signature to predict the 1-year and 3-year overall survival (OS) of HCC patients. Transcriptome and clinical survival information of HCC patients was obtained from The Cancer Genome Atlas database. A set of ARlncRNAs was identified by co-expression analysis, from which seven ARlncRNAs (AC005229.4, AL365203.2, AL117336.3, AC099850.3, ELFN1-AS1, LUCAT1, and AL031985.3) were selected for use as a predictive signature. Risk scores were derived for each patient, who were then divided into high-risk and low-risk groups according to the median risk value. The OS of high-risk patients was significantly lower than that of low-risk patients (*P* < 0.0001). The 1- and 3-year time-dependent ROC curves were used to evaluate the predictive ability of the risk score (AUC = 0.785 of 1 year, 0.710 of 3 years), and its predictive ability was found to be better than TNM stage. Moreover, the risk score was significantly, linearly related to pathological grade and TNM stage (*P* < 0.05). Overall, a novel nomogram to predict the 1-year and 3-year OS of HCC patients was developed, which shows good reliability and accuracy, for use in improved treatment decision-making.

## Introduction

Hepatocellular carcinoma (HCC) is a tumor of the digestive system with high malignancy, high rate of metastasis and recurrence, and poor prognosis. It is the most common pathological type of primary liver cancer, the fifth most common malignant tumor worldwide, and the second leading cause of cancer-related deaths (Bray et al., 2018). Asia and Africa exhibit a high incidence of HCC, which has gradually increased over recent years (Ozakyol, 2017). Hepatitis virus infection, alcohol, aflatoxin, metabolic syndrome, and other risk factors are closely related to the occurrence of HCC (Bruix and Sherman, 2005). Surgical resection and liver transplantation of early HCC patients can effectively control tumor development and prolong survival time (Padhya et al., 2013). However, HCC has an insidious onset and rapid progression. Many patients have reached the mid-to-late stages when they are diagnosed, and the chances of surgical resection are limited. Interventional therapy, local ablation, radiotherapy, targeted therapy, and other measures have limited satisfactory outcomes, and the poor prognosis of HCC remains (Huynh, 2010). Therefore, exploring potential molecular mechanisms and cell signaling pathways underlying the pathogenesis of HCC, studying markers with prognostic value, and constructing a prognostic signature will improve clinical decision-making, benefiting patients and extending the prognostic survival time.

Autophagy is a biological process that degrades senescent and damaged cytoplasmic components (proteins and organelles) by lysosomes under the regulation of autophagy-related genes (ARGs). It is considered a self-protection mechanism in eukaryotes, and is often activated in cells to resist damage, inflammation, and tumor development, maintain cellular homeostasis, balance cell metabolism, and adapt to environmental changes (Graef, 2020). In 2016, the Nobel Prize in Physiology or Medicine was awarded to Japanese scientist Yoshinori Ohsumi for his pioneering research in elucidating the molecular mechanism and physiological functions of autophagy, and research on autophagy has since received increasing attention (Mizushima, 2017). Autophagy is not only involved in the physiological processes of the body, but also closely related to the occurrence and development of neurodegenerative diseases, tumors, and other diseases (Saha et al., 2018; Yun and Lee, 2018). For tumors, autophagy is a double-edged sword. In the process of tumor initiation, autophagy inhibits the transformation of normal cells into tumor cells by removing damaged proteins, DNA, and necrotic organelles. In the later stage, autophagy provides a large amount of nutrients and energy for tumor cells with a strong metabolism, so that tumor cells can withstand the harsh tumor microenvironment, resist the pressure of chemotherapy or radiotherapy, and help tumor cells to survive and grow (Tiessen et al., 2019). Many studies have shown that dysregulation of the autophagy pathway is involved in the pathophysiological processes of liver steatosis, chronic hepatitis, liver fibrosis, and HCC (Gual et al., 2017). Research on the relationship between HCC and autophagy is gaining momentum, and the potential value of autophagy as a therapeutic target and prognostic indicator for patients with HCC is currently being explored.

Long non-coding RNA (lncRNA) is a type of RNA sequence with a length greater than 200 nucleotides and no protein coding function. lncRNAs participate in regulating gene expression at the transcription level, post-transcriptional level, and translation level, and also plays an important role in numerous cellular activities, such as dosage compensation effect, epigenetic regulation, cell cycle regulation, and cell differentiation regulation (St Laurent et al., 2015). It has been reported that dysregulation of the expression and function of lncRNAs is involved in the occurrence and development of malignant tumors, including tumor cell proliferation, invasion, metastasis, and chemotherapy resistance (Liu et al., 2018; Xu et al., 2018). By comparing tumor tissues and normal tissues, it was found that there are differences in the expression of multiple lncRNAs in HCC (Lanzafame et al., 2018), and some lncRNAs can regulate ARGs at the transcriptional and post-transcriptional levels, thereby participating in the autophagy pathway (Yang et al., 2016; Jing et al., 2020). Although most of the functions of lncRNAs remain to be elucidated, we can explore possible biological functions through lncRNA-mRNA co-expression analysis. In this study, co-expression network analysis of differentially expressed ARGs (DEARGs) and differentially expressed lncRNAs (DElncRNAs) will be used to identify for differentially expressed autophagy-related lncRNAs (DEARlncRNAs), to construct a prognostic signature to evaluate the prognosis of HCC patients.

## Materials and Methods

### Data Acquisition

The transcriptome sequencing data, survival information, and clinical information of HCC patients was downloaded from The Cancer Genome Atlas (TCGA)^1^. The list of ARGs was downloaded from the Human Autophagy Database (HADb) website^2^.

### Identification of Differentially Expressed mRNAs and lncRNAs in HCC and Normal Tissues

The transcriptome sequencing data was divided into protein encoding mRNA and lncRNA expression data, and the expression data of ARGs was derived from the protein encoding mRNA expression data. The R language “limma” package was used to analyze the DEARGs and DElncRNAs in HCC tissues and normal tissues. The screening criteria were set to | log_2_ Fold Change (FC)| > 1 and FDR < 0.05. The “ggpot2” package was used to draw the volcano plots of DEARGs and DElncRNAs. Heatmaps of the differentially expressed genes were plotted by the “pheatmap” package.

### Construction of a Co-expression Network Between DEARGs and DElncRNAs

By using the “limma” package, *Pearson* correlation was used to calculate the correlation between the expression of DEARGs and DElncRNAs in HCC patients. A DElncRNA with Pearson correlation coefficient (PCC) >0.4 and *P* < 0.001 was considered to be a DEARlncRNA. Then, we obtained a list of DEARlncRNAs through the construction of the co-expression network.

### Identification of DEARlncRNAs With Prognostic Value

Univariate Cox regression analysis was used to explore the prognostic value of DEARlncRNAs in HCC patients with the “survival” package in R, and the genes with significant overall survival (OS) differences among them were identified (*P* < 0.05). Subsequently, LASSO regression analysis was performed to screen the significantly OS-related DEARlncRNAs by the “glmnet” package. The optimal value for penalization coefficient lambda (λ) was selected by running cross-validation likelihood 1,000 times. This method could avoid overfitting of the signature. While obtaining the minimum λ, the lncRNAs that were the most suitable for building the signature were selected.

### Construction and Evaluation of the Prognostic Signatures of ARlncRNAs

Multivariate Cox regression analysis was performed on the DEARlncRNAs screened by LASSO. Through trying different combination of lncRNAs to construct signatures and calculating the Akaike information criterion (AIC) value of each one, the best prognosis signature would be selected with the minimum AIC value which had goodeness of fit. The risk score calculation formula was used to calculate the risk score for each patient: risk score = coef gene 1 × gene 1 expression + coef gene 2 × gene 2 expression + . + coef gene Ñ × gene Ñ expression. The risk score was obtained by weighting the expression level of lncRNA and the regression coefficient (coef). The coef value was calculated by log transformation of the hazard ratio (HR) from the multivariate Cox regression analysis, and each expression of lncRNA involved in the prognosis signature was defined as gene Ñ expression. Based on the median risk score, all HCC patients from TCGA were divided into high-risk and low-risk groups.

Firstly, the expression differences of lncRNAs in the signature were identified by paired *t-test* in 50 pairs of HCC tumor tissues and adjacent normal tissues from TCGA database. The Kaplan–Meier (K-M) method was used to analyze the OS difference between the high- and low-risk groups, and a survival curve was drawn. We drew 1- and 3-year time-dependent Receiver Operating Characteristic (ROC) curves by “survivalROC” package, compared the predictive ability of risk score and other clinical features, and evaluated the sensitivity and specificity through the area under the curve (AUC value). In order to verify whether the ARlncRNA risk score could be used as an independent predictor for the prognosis of HCC patients, age, sex, pathological stage, clinical stage, and risk score were included in the univariate and multivariate Cox regression analysis.

### Analysis of Risk Scores and Clinical Features of HCC Patients

Sex (male, female), age (<60, ≥60 years), tumor pathological grade (G1, G2, and G3 ∼ 4), and TNM stage (Stage I, Stage II, and Stage III ∼ IV) of patients in the high- and low-risk group were compared, to determine whether there were significant differences in risk scores between different groups. Then, compared whether there were significant differences in OS between high-and low-risk patients when they had the same clinical features by using K-M method. According to the quartiles (*Q*) of all risk scores, the patients were divided into higher-risk patients (risk scores ≥ *Q3*) and lower-risk patients (risk scores ≤ *Q1*). The K-M method was used to compare the survival differences between the two groups.

Gene set enrichment analysis (GSEA) can be used to determine whether the gene set is statistically significantly enriched in some functional pathways between the two groups. In this study, we analyzed the Kyoto Encyclopedia of Genes and Genomes (KEGG) pathways enriched by the genes that were expressed in high-risk patients. The pathways with normalized enrichment score (NES > 1, *P* < 0.01, and FDR < 0.05) were screened as significantly enriched pathways.

### Construction and Validation of Nomogram Based on Risk Score

The nomogram is a graphical display of the regression equation, which is intuitive, simple, and easy to clinical application. Screened the factors that affected the prognosis of HCC patients, and used the “survival” and “rms” packages to construct a nomogram based on the Cox regression to predict the 1-year and 3-year survival probability of HCC patients. Then we used the internal validation method to test the predictive ability of the nomogram. The Bootstrap self-sampling method was repeated 1,000 times, and we calculated the concordance index (C-index) to compare the consistency of the predicted results with the actual results. In order to evaluate the accuracy of the nomogram prediction, the difference between the predicted results of the nomogram and the actual results were displayed in the calibration curves. Finally, drew the time-dependent ROC curves of the nomogram and calculated the AUC values.

### Statistical Methods

**FIGURE 1:**
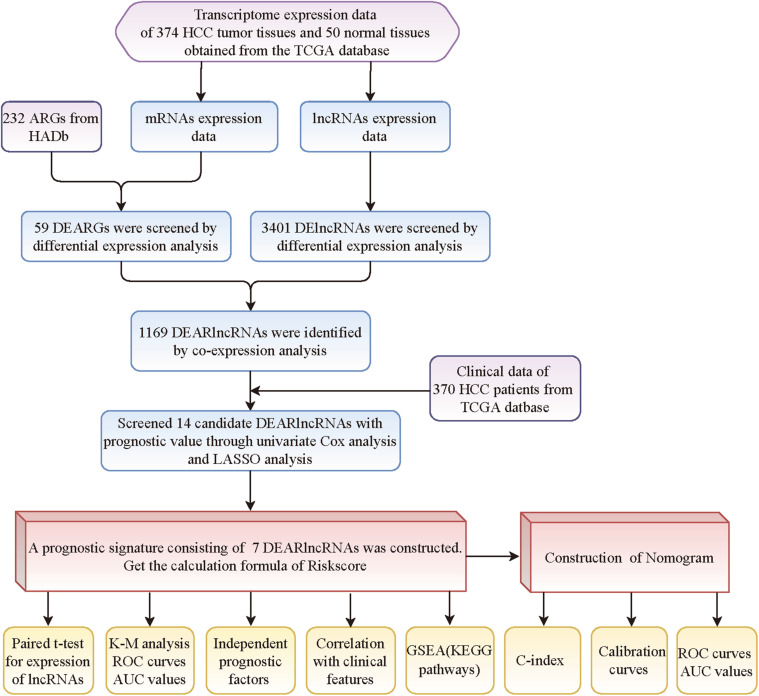
The flowchart of this study.

Figure 1 summarizes the flowchart of this study. The content of this study was completed using R software (v3.6.3), GraphPad Prism (v8.0), and GSEA software (4.1.0). The Wilcox test was used to analyze the difference in gene expression between tumor and normal tissues. Univariate and multivariate Cox analyses were used to evaluate the relationship between lncRNA expression and survival of HCC patients, and the relationship between risk score and clinical information and the prognosis of HCC patients. The K-M method was used to compare the OS difference between the high-risk and low-risk groups, and the log-rank test was used to calculate the *P* value. The *t*-test was used compare the differences in risk score among different clinical feature groups. *P* < 0.05 is considered statistically significant.

## Results

### Identification of DEARlncRNAs in HCC and Normal Tissues

**FIGURE 2:**
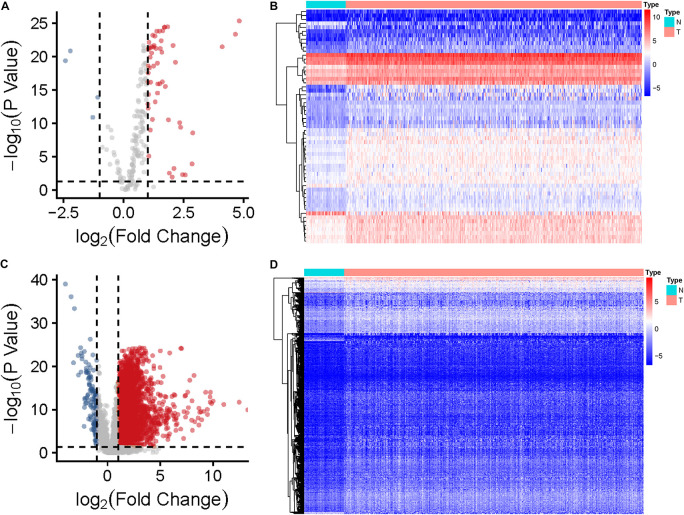
Identification of DEARGs and DElncRNAs in HCC and normal tissues. **(A)** Volcano plots of the distributions of 59 DEARGs (the red dots indicate up-regulated ARGs and the blue dots indicate the down-regulated ARGs). **(B)** Heatmap of the expression profile of DERGs in HCC and normal tissues. **(C)** Volcano plots of the distributions of 3,401 DElncRNAs. **(D)** Heatmap of the expression profile of DElncRNAs in HCC and normal tissues.

The expression data of 374 HCC tissues and 50 normal tissues were downloaded from TCGA database, and data of 370 HCC patients with expression data, survival status, and clinical information were also collated. A list of 232 autophagy genes was downloaded from the HADb website, and 194 of them were identified as being expressed in the liver. Using|log_2_ Fold Change (FC)| > 1 and FDR > 0.05 as the screening criteria, the DElncRNAs and DEARGs was calculated. As a result, 59 DEARGs and 3,401 DElncRNAs were obtained (Figures 2A–D). These differentially expressed genes were subjected to co-expression analysis, and the *Pearson* correlation coefficient (*PCC*) > 0.4 and *P* < 0.001 were used as the screening criteria, and 303 lncRNAs significantly related to ARGs were obtained (Supplementary Material 1). These lncRNAs were used as DEARlncRNAs.

### Construction of the DEARlncRNA Prognostic Signature for HCC Patients

**FIGURE 3:**
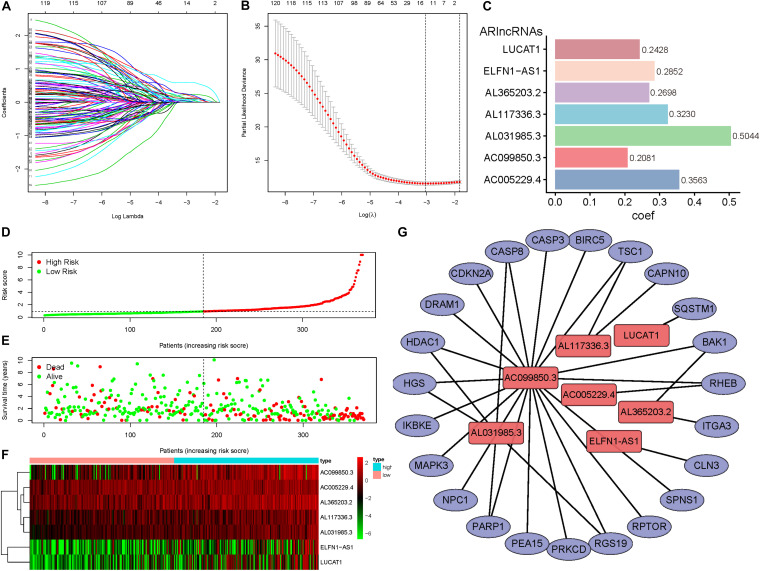
Construction of the DEARlncRNAs prognostic signature for HCC patients. **(A,B)** LASSO analysis was performed on 121 DEARlncRNAs with prognostic value selected by univariate Cox analysis, and 14 best candidate DEARlncRNAs were obtained for constructing a prognostic signature.**(C)** The multivariate Cox analysis of the 14 DEARlncRNAs previously screened resulted in a prognostic signature composed of seven DEARlncRNAs. The coef value of each lncRNA expression in the risk score calculation formula was the regression coefficient of the prognostic signature, and barchart reflected the coef value. **(D)** Distribution of risk scores of high- and low-risk HCC patients. **(E)** Scatter plot shows the correlation between survival time and risk score. **(F)** The heatmap of the seven DEARlncRNAs expression profiles in high- and low-risk HCC patients.**(G)** Network shows the co-expression relationship between the seven DEARlncRNAs and their corresponding ARGs.

The 303 DEARlncRNAs were subjected to univariate Cox regression analysis with the survival information of 370 HCC patients, and 121 lncRNAs were found to have significant prognostic differences (*P* < 0.05) (Supplementary Material 2). When the minimum value of λ was 0.0478, LASSO analysis revealed the 14 best candidate DEARlncRNAs which could reduce overfitting of the signature, including NRAV, ZFPM2-AS1, PRRT3-AS1, AC005229.4, AL365203.2, AL117336.3, AC007405.3, AC099850.3, ELFN1-AS1, DANCR, LUCAT1, AL031985.3, AC009005.1, and MIR210HG (Figures 3A,B).

The 14 DEARlncRNAs with prognostic significance were subjected to multivariate Cox analysis to construct a prognostic signature. According to the minimum AIC value (AIC = 1281.3), a prognostic signature consisting of seven lncRNAs was obtained, consisting of AC005229.4, AL365203.2, AL117336.3, AC099850.3, ELFN1-AS1, LUCAT1, and AL031985.3; all of these genes were risk factors for prognosis (Hazard Ratio > 1). Based on the prognosis signature, the risk score calculation formula was obtained: risk score = (0.3563 × AC005229.4 expression) + (0.2698 × AL365203.2 expression)+ (0.3230 × AL117336.3 expression) + (0.2081 × AC099850.3 expression) + (0.2852 × ELFN1-AS1 expression) + (0.2428 × LUCAT1 expression) + (0.5044 × AL031985.3 expression) (Figure 3C). The risk score of each HCC patient was calculated based on the expression levels of seven lncRNAs, and the patients were divided into high-risk group (*n* = 185) and low-risk group (*n* = 185) by the median value (risk score = 0.9108). Figure 3D shows the distribution of the risk score of the HCC patients, and the risk scores gradually increased from left to right, and the patients were divided into two groups. Figure 3E shows the distribution of survival status and survival time of patients with different risk scores. A heatmap was constructed to show the expression of ARlncRNAs in the high- and low-risk groups (Figure 3F), the expression of the seven lncRNAs in high-risk patients was higher than those in low-risk patients. The network showed the co-expression relationship between DEARGs and DEARlncRNAs which constituted the prognosis signature, and all relationships satisfied the criteria of *PCC* > 0.4 and *P* < 0.001 (Figure 3G).

### Validation of the Predictive Ability of Seven DEARlncRNA Prognostic Signatures

**FIGURE 4:**
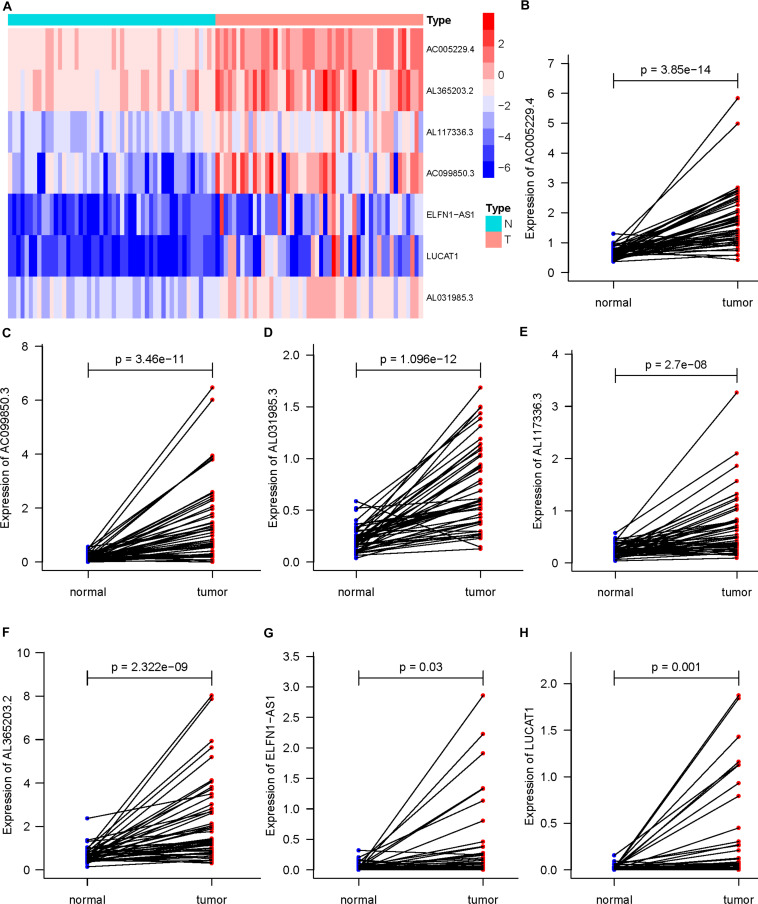
Differential expression of seven DEARlncRNAs in 50 pairs of HCC tumor tissues and adjacent normal tissues (*P* < 0.05). **(A)** The heatmap of expression; **(B)** AC005229.4; **(C)** AC099850.3; **(D)** AL031985.3; **(E)** AL117336.3; **(F)** AL365203.2; **(G)** ELFN1-AS1; **(H)** LUCAT1.

**FIGURE 5:**
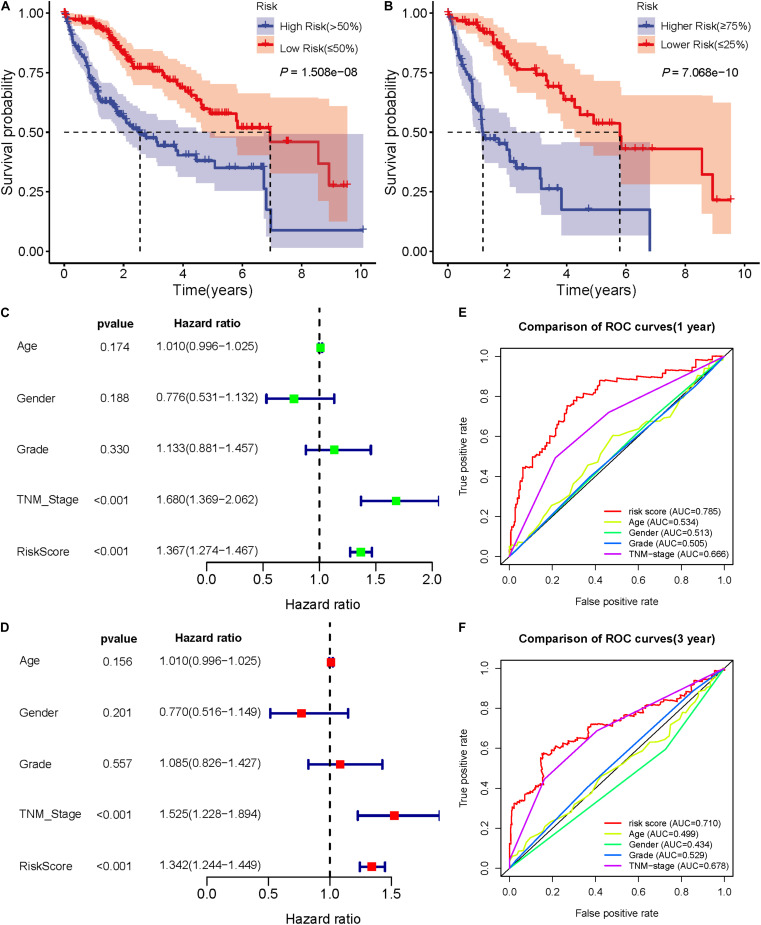
Validation of the prognostic signature of seven DEARlncRNAs. **(A)** The K-M curve reflects that the OS of high-risk HCC patients is significantly lower than that of low-risk patients (*P* < 0.0001). **(B)** The K-M curve reflects that the OS of higher-risk patients is also significantly worse than that of lower-risk patients (*P* < 0.0001). **(C)** Forest plot reflects the univariate Cox analysis of the relationship between the clinical features, risk score and OS of HCC patients. Both TNM stage and risk score significantly affect the prognosis of HCC patients (*P* < 0.001).**(D)** Forest plot reflects multivariate Cox analyzed the relationship between the clinical features, risk score and OS of HCC patients. TNM stage and risk score are independent prognostic risk factors for HCC (*P* < 0.001). **(E)**The 1-year time-dependent ROC curve shows that the prediction accuracy of risk score is higher than other clinical features (AUC = 0.785). **(F)** The 3-year time-dependent ROC curve reflects that the prediction accuracy of risk score is higher than other clinical features (AUC = 0.710).

In 50 pairs of HCC tumor tissues and adjacent normal tissues, we drew a heatmap to show the expression of seven lncRNAs (Figure 4A). Then, we confirmed that there were significant differences in the expression of seven lncRNAS by paired *t*-test, and the expression in tumor tissues was significantly higher than that in normal tissues (*P* < 0.05) (Figures 4B–H). A K-M curve was used to compare the OS time difference between the high- and low-risk groups (Figure 5A). The OS of HCC patients with high-risk scores was significantly lower than that of patients with a low-risk score (*P* < 0.0001), which is consistent with the results of the prognosis signature. Through the quartiles (*Q*) of the risk scores, patients were divided into higher-risk groups (risk scores ≥ *Q3, Q3* = 1.479) and lower-risk groups (risk scores ≤ *Q1, Q1* = 0.595), and then the K-M curve was drawn to find that the OS of higher-risk group was also significantly worse than that of lower-risk group (*P* < 0.0001) (Figure 5B).

Univariate and multivariate Cox analysis was used to investigate whether sex, age, pathological grade, TNM stage, and risk score of HCC patients were independent prognostic factors. The forest plot results (Figures 5C,D) show that TNM stage and risk score were both independent prognostic factors (*P* < 0.001), which could affect the prognosis of HCC patients. Time-dependent ROC curves were drawn to predict the 1-year and 3-year survival rates of HCC patients, which showed that the risk score has good sensitivity and specificity. The 1-year AUC = 0.785 and the 3-year AUC = 0.710 of risk score, which were higher than the AUC values of sex, age, pathological grade, and TNM stage (Figures 5E,F). These showed that the predictive results of the prognostic signature are reliable and accurate.

### Correlation Analysis Between the Risk Scores and Clinical Features of HCC Patients

**FIGURE 6:**
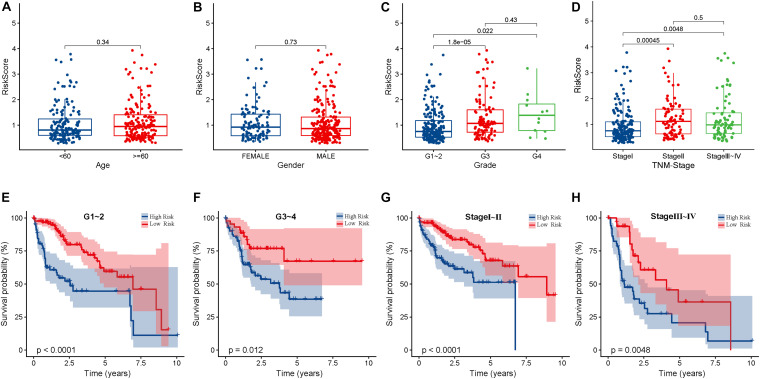
The correlation between the clinical features and risk score of HCC patients. **(A)** There is no significant difference between risk scores and different ages (<60,≥60 years old) (*P* > 0.05). **(B)** There is no significant difference difference between risk score and different genders (*P* > 0.05). **(C)** Pathological grade and risk score are correlated. HCC patients with different pathological grades have significant differences in risk score, G1 ∼ 2 vs G3 (*P* < 0.05), G1 ∼ 2 vs G4 (*P* < 0.05), but G3 vs G4 (*P* > 0.05). **(D)** TNM stage and risk score are correlated. There are significant differences in risk score of HCC patients in different stages, Stage I vs Stage II (*P* < 0.05), Stage I vs Stage III ∼ IV (*P* < 0.05), but Stage II vs Stage III ∼ IV (*P* > 0.05). **(E)** In patients with pathological grade G1 ∼ G2, the OS of high-risk patients was significantly lower than that of low-risk patients (*P* < 0.05). **(F)** In patients with pathological grade G3 ∼ G4, the OS of high-risk patients was significantly lower than that of low-risk patients (*P* < 0.05). **(G)** In patients with TNM stage I ∼ II, the OS of high-risk patients was significantly lower than that of low-risk patients (*P* < 0.05). **(H)** In patients with TNM stage III ∼ IV, the OS of high-risk patients was significantly lower than that of low-risk patients (*P* < 0.05).

The risk score was found to be not significantly correlated to sex and age (*P* > 0.05), but significantly correlated to pathological grade and TNM stage, G1 ∼ 2 vs. G3 (*P* < 0.05), G1 ∼ 2 vs. G4 (*P* < 0.05), and Stage I vs. Stage II (*P* < 0.05), Stage I vs. Stage III ∼ IV (*P* < 0.05). Thus we speculate, as the pathological grade and TNM stage increase, the risk score may also be higher, which indicates a worse prognosis (Figures 6A–D). In particular, G3 vs. G4 (*P* > 0.05) and Stage II vs. Stage III ∼ IV (*P* > 0.05), showed no significant difference between these groups, and we need to increase the sample capacity to continue to verify their correlation. K-M curves showed that patients with higher pathological grade, higher TNM stage, and high risk scores might have worse prognosis (Figures 6E–H). In addition, the higher the risk score, the worse the prognosis in patients with the same pathologic grade or same TNM stages (*P* < 0.05).

### Enrichment Analysis of GSEA-KEGG Pathway in High-Risk Patients

**FIGURE 7:**
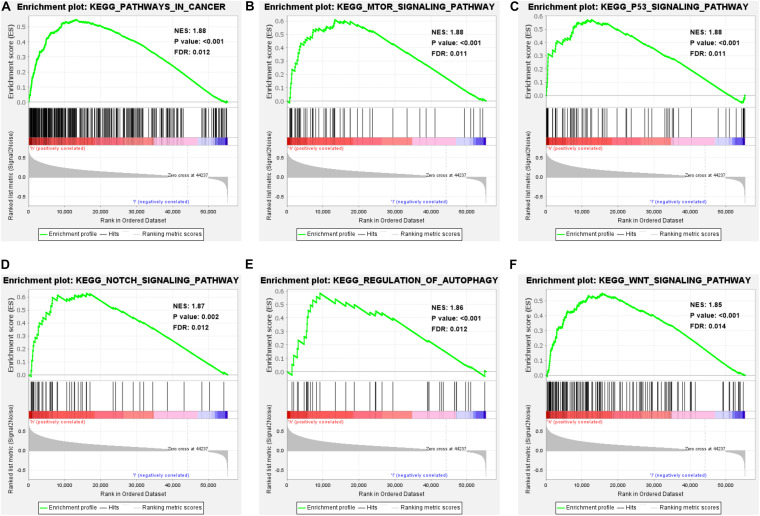
Enrichment analysis of GSEA-KEGG pathway in high-risk patients. **(A–F)** Performing GSEA on HCC patients showed that the genes expressed by high-risk patients were significantly enriched in pathways in cancer, regulation of the autophagy pathway, mTOR signaling pathway, p53 signaling pathway, Notch signaling pathway, and Wnt signaling pathway (NES > 1, *P* < 0.05, and FDR < 0.05).

GSEA shows the enrichment of KEGG pathways in high-risk HCC patients. We know that the genes expressed by high-risk patients are enriched in multiple important pathways (NES > 1, *P* < 0.01, and FDR < 0.05), including pathways in cancer, regulation of the autophagy, the mTOR signaling pathway, the p53 signaling pathway, the Notch signaling pathway, and the Wnt signaling pathway (Figures 7A–F). In addition, the genes enriched in each significant pathway can be presented in Supplementary Material 3. Notably, genes involved in the regulation of autophagy pathway were significantly enriched in patients with high risk scores (NES = 1.86, *P* < 0.001, and FDR = 0.012). These results not only indicate that the high risk score is associated with autophagy and cancer processes, but also offers important insight into the pathways highly enriched in HCC patients, which could serve as potential therapeutic targets.

### Construction and Validation of the Nomogram

**FIGURE 8:**
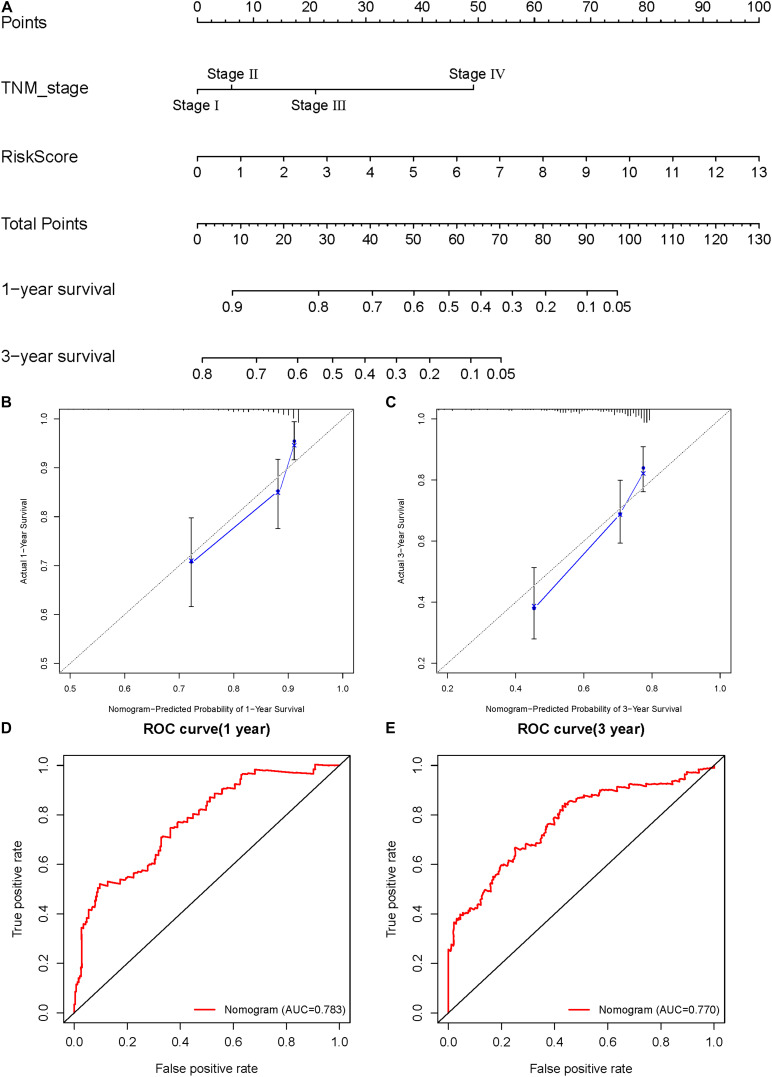
Construction and validation of the nomogram. **(A)** Calculate the scores of each item of HCC patients according to the nomogram, and the total scores obtained after addition can predict the 1- and 3-year survival probability. **(B,C)** The 1- and 3-year calibration curves of the nomogram. **(D,E)** The ROC curves of 1-and 3-year nomogram (AUC = 0.783 of 1 year, AUC = 0.770 of 3 years).

A nomogram was constructed to assist in clinical interpretation of the predictive signatures to conveniently determine the survival rate of HCC patients. Based on the previous Cox regression analysis of independent prognostic factors, we knew that TNM stage and risk score were both independent prognostic factors. So, we used these two factors to construct a nomogram (Figure 8A). According to the nomogram, the score of each item for the HCC patients must be calculated and summed to obtain the total score, which will predict the survival probability of 1 and 3 years, which is conducive in guiding clinical decision-making. Based on internal validation by Bootstrap self-sampling, the C-index of the nomogram was 0.734, indicating that it has an accurate predictive ability. The calibration curves of the nomogram show that the 1- and 3-year predicted survival rates have good accuracy (Figures 8B,C). The closer the calibration curve is to the diagonal, the more accurate the prediction result is. The ROC curves of 1 and 3 years also show that the predictive ability of the nomogram is very accurate (AUC = 0.783 of 1 year, AUC = 0.770 of 3 years) (Figures 8D,E). The nomogram demonstrates reliable and convenient clinical application.

## Discussion

Hepatocellular carcinoma is one of the most common malignant tumors affecting humans worldwide. Although the continuous development and advancement of surgical techniques in recent years have improved HCC prognosis, the treatment options available for patients with advanced HCC are limited and typically ineffective. Therefore, exploring the pathogenesis of HCC and seeking new targets for diagnosis and targeted therapy, will allow for the establishment of an effective prognostic signature that may help guide the formulation of individual treatment strategies.

In this study, we analyzed the transcriptome information and clinical data of HCC patients in TCGA database, and found a set of DEARlncRNAs through co-expression analysis, of which 121 lncRNAs were found to have prognostic significance through univariate Cox analysis. Multivariate Cox analysis allowed for the construction of a new prognostic signature based on seven DEARlncRNAs, including AC005229.4, AL365203.2, AL117336.3, AC099850.3, ELFN1-AS1, LUCAT1, and AL031985.3. According to the prognosis signature, the risk score of each patient can be calculated, which is an independent risk factor affecting the prognosis, and can be used to predict the OS of HCC patients. High-risk patients have a worse prognosis than low-risk patients. Analyzing the relationship between the risk score and clinical characteristics, we concluded that the risk score is significantly related to the pathological grade and TNM stage of the tumor. The higher the pathological grade and the later TNM stage of the tumor, the higher the risk score and the worse the prognosis. We therefore speculate that the seven DEARlncRNAs that comprise the prognostic signature are involved in the progression of HCC. By analyzing the ROC curves of HCC patients′ 1-year and 3-year survival, we found that the prognostic signature has good sensitivity and specificity (AUC = 0.785, 0.710), and can be used to reliably predict the prognosis of HCC patients. In order to facilitate clinical application and obtain more accurate prediction results, we finally constructed a nomogram to predict the 1-year and 3-year survival rates of patients. According to the C-index, calibration curves and ROC curves, the nomogram has a good prediction accuracy, and the predicted results are in good agreement with the real results.

In previous studies, the autophagy pathway was thought to be involved in HCC occurrence, progression, and chemotherapy resistance (Amaravadi et al., 2011; Levine and Kroemer, 2019). Autophagy is a highly conserved process, and its normal function depends on a series of ARGs. lncRNAs have been shown to regulate autophagy by directly affecting the expression of ARGs, acting as competitive endogenous RNAs (ceRNAs) to regulate miRNAs, affect the expression of downstream ARGs, and interact with different regulatory pathways, which impacts tumor occurrence and development (Sun, 2018). The lncRNA HULC is highly expressed in liver cancer cells. Xin et al. (2018) showed that HULC can promote the occurrence and development of liver cancer by inhibiting the autophagy-related gene PTEN, regulating ATG3, and reducing the sensitivity of HCC cells to chemotherapy drugs (Xiong et al., 2017). Liu et al. (2016) found that the lncRNA HNF1A-AS1 is significantly highly expressed in HCC cells, which acts on miR-30b to upregulate the expression of ATG5 and ATG12, promotes autophagy, and ultimately promotes the occurrence and development of liver cancer. The lncRNA MEG3 is considered to be a tumor suppressor gene, which can reduce autophagy and inhibit the proliferation of liver cancer and other tumor cells. MEG3 is significantly low in liver cancer tissues, which is closely related to chemotherapy resistance and poor prognosis (Pu et al., 2019). It is evident that lncRNAs can regulate the process of autophagy and participate in the occurrence, development, invasion, metastasis, and chemotherapy resistance of HCC, and affect the survival of HCC patients. However, few researchers have systematically studied the prognostic value of autophagy-related lncRNAs. Hence, we constructed a prognostic signature based on autophagy-related lncRNAs to predict the prognosis of HCC patients.

Currently, only a small part of the large family of lncRNAs has been studied. Among the seven ARlncRNAs selected here, ELFN1-AS1 has also been found to be highly expressed in colon and ovarian tumor tissues, where it promotes the proliferation, invasion, and metastasis of tumor cells, and is related to poor prognosis (Jie et al., 2020; Lei et al., 2020). However, its role in the autophagy pathway has not been reported yet. LUCAT1 is highly expressed in HCC tissues, which can promote tumor cell proliferation and metastasis, and is an independent risk factor for poor survival in HCC patients (Jiao et al., 2019; Lou et al., 2019). The study of Shen et al. (2020) showed that LUCAT1 regulated the sensitivity of tumor cells to cisplatin by upregulating autophagy-related gene ULK1 *via* sponging miR-514a-3p in non-small cell lung cancer. The other five lncRNAs have not been found in relevant studies to report their functions or pathways involved. However, according to the co-expression network we constructed, we know that AC099850.3 has co-expression relationships with 19 ARGs, and AL031985.3 has co-expression relationships with five ARGs. We speculate that these genes are likely to participate in the autophagy pathway. The functions of most lncRNAs and their roles in tumors are still unknown. In this study, the lncRNAs selected by co-expression analysis, their function in the autophagy pathway as well as their role in the occurrence and development of liver cancer through autophagy, remains to be experimentally studied. We believe that this study will provide the foundation and basis for further investigations.

We conducted GSEA of the genes expressed by HCC patients with high risk scores. The results showed that the genes highly expressed in high-risk patients were not only enriched in the autophagy regulatory pathway, but also in many well-known tumor pathways related to occurrence and development, including the mTOR signaling pathway, p53 signaling pathway, Wnt signaling pathway, and Notch signaling pathway. The promotion of these pathways in the occurrence, development, angiogenesis, invasion, and metastasis of HCC has been confirmed by many studies (Zucman-Rossi et al., 2015; Huang et al., 2019). The significant enrichment of genes involved in the signal transduction of these pathways also indicates that high-risk patients are more likely to have disorders of these pathways that will potentially contribute to tumor progression. The initiation of autophagy is complex and involves numerous ARGs and multiple signaling pathways. Autophagy and different signaling pathways are largely related, forming a huge regulatory network, which ultimately affects the fate of tumor cells. Investigation into these pathways will offer insight into the molecular mechanisms underlying HCC and may provide potential therapeutic targets.

Inevitably, this study has certain limitations. First, ARlncRNAs are identified by constructing a co-expression network. Their function and molecular involvement in the process of autophagy in HCC needs to be further experimentally studied. Second, the sample capacity should be expanded before proceeding with further correlative studies between the risk score and clinical features (such as pathological grade and TNM stage). Third, this study is a retrospective study based on TCGA database. The prognostic signature and nomogram need to be validated in prospective clinical studies to test their predictive effect and accuracy.

In summary, this study established an ARGs-lncRNA co-expression network, identified seven DEARlncRNAs that have prognostic significance, and constructed a new prognostic signature, which has good sensitivity and specificity for predicting the 1- and 3-year survival rate of HCC patients. The nomogram contains the risk score derived from the prognostic signature and some clinical features. According to the total score, the survival rate of HCC patients at 1- and 3-years can be predicted, which helps guide clinicians to formulate personalized treatment plans and thus improve the quality of care.

## Data Availability Statement

The datasets analyzed for this study can be found in The Cancer Genome Atlas (https://portal.gdc.cancer.gov/) and the Human Autophagy Database (http://www.autophagy.lu/).

## Author Contributions

JL and YJ: conception and design. YJ and YC: acquisition, analysis, and interpretation of data. YJ and YC: figures drawing. YJ and JL: writing and revision of manuscript. JL: study supervision. All authors read and approved the final manuscript.

## Conflict of Interest

The authors declare that the research was conducted in the absence of any commercial or financial relationships that could be construed as a potential conflict of interest.

## Footnotes

1 https://portal.gdc.cancer.gov/

2 http://www.autophagy.lu/

## Supplementary Material

Supplementary Material 1 Identification of 303 DEARlncRNAs through co-expression analysis.

Supplementary Material 2 The forest plot of 121 DEARlncRNAs with significant prognostic value.

Supplementary Material 3 Details of the significantly enriched GSEA-KEGG pathways in high-risk patients.
