# Supplementary figures and images for: Prognosis-Predictive Signature and Nomogram Based on Autophagy-Related Long Non-coding RNAs for Hepatocellular Carcinoma
Source: Front Genet. 2020 Dec 23;11:608668. doi: 10.3389/fgene.2020.608668 (PMC7793718; doi:10.3389/fgene.2020.608668)

Supplementary Material 2:

The forest plot of 121 DEARlncRNAs with significant prognostic value

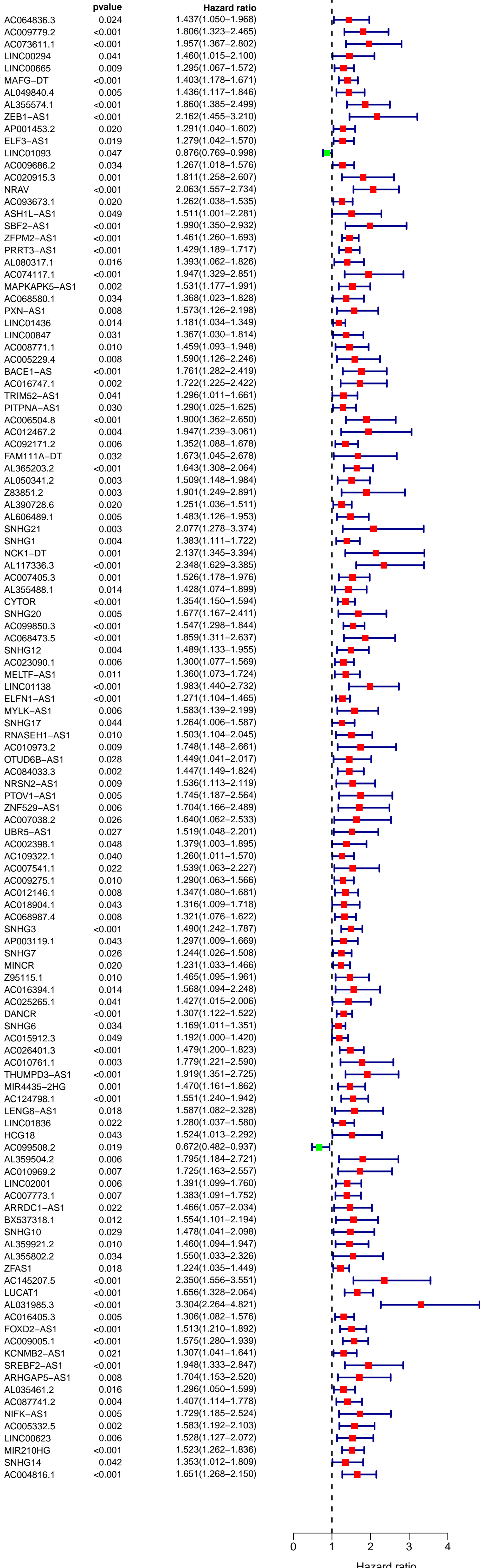

Supplement: Supplementary Material 2 — The forest plot of 121 DEARlncRNAs with significant prognostic value. [file Data_Sheet_2.pdf]
