# Supplementary material for: Prognosis-Predictive Signature and Nomogram Based on Autophagy-Related Long Non-coding RNAs for Hepatocellular Carcinoma
Source: Front Genet. 2020 Dec 23;11:608668. doi: 10.3389/fgene.2020.608668 (PMC7793718; doi:10.3389/fgene.2020.608668)
Supplement: Supplementary Material 3 — Details of the significantly enriched GSEA-KEGG pathways in high-risk patients. [file Data_Sheet_3.pdf]

Supplementary Material 3：

Details of the significantly enriched GSEA-KEGG pathways in high-risk patients

KEGG Pathways	NES	*P* value	FDR	Core enriched genes
PATHWAYS_IN_CANCER	1.88	<0.001	0.012	MSH2,DVL3,TPR,CCNE2,TRAF1,MAX,TGFBR1,GLI1,HSP90AA1,SOS2, E2F3,RHOA,PIAS2,PIK3CB,NFKB2,NFKB1,FZD7,TRAF6,RASSF5,TCF7, GRB2,BRCA2,CDKN2A,CTBP1,PTK2,HIF1A,RALGDS,SMAD4,ELOC,CEBPA, NRAS,CUL2,TFG,SMAD3,EGLN3,PIAS1,BID,PTCH1,FADD,PIK3CD, TPM3,MAPK1,GSK3B,RAF1,SKP2,SUFU,XIAP,LEF1,FZD1,FGFR1, VHL,APPL1,MSH6,FZD6,MAPK8,BIRC2,E2F1,CSF2RA,WNT5A,CXCL8, E2F2,CDC42,ITGAV,VEGFA,PRKCA,RALBP1,JAK1,MAP2K2,BIRC3,FGF11, CDK4,SMAD2,TRAF2,KITLG,PIK3CA,FZD3,MDM2,RALB,HRAS,SOS1, STK4,TRAF5,MAPK9,CKS1B,ITGB1,LAMB1,ITGA3,DAPK3,CSF3R,RXRB, RAD51,DVL2,ABL1,BRAF,CASP3,CBLB,PPARD,FN1,TP53,ITGA6, CASP8,AXIN1,ARNT2,RELA,EP300,EGF,DVL1,RB1,LAMA4,RAC2, CDK2,MAPK3,STK36,LAMC1,RUNX1,BAX,CHUK,SPI1,FZD2,TGFB2, RALA,CCDC6,CDKN2B,PIAS4,IKBKB,LAMA5,TGFB1,STAT5B,APC,AKT2, HDAC2,CBL,PLCG1,CTNNA1,CREBBP,MECOM,BCR,CTNNB1,CTBP2,VEGFB, RASSF1,MLH1,CRKL,SLC2A1,RBX1,IGF1R,KRAS,BCL2L1,FZD5,ARNT, BIRC5,HSP90AB1,HDAC1,PML,ITGA2,TRAF4,FGFR3,MAP2K1,PPARG,MTOR, PIAS3,TRAF3,RAC1
MTOR_SIGNALING_PATHWA Y	1.88	<0.001	0.011	STRADA,MAPK1,RHEB,RPS6KB1,RPTOR,TSC1,MAPK3,RICTOR,EIF4E,PIK3CB, VEGFA,CAB39,RPS6KA3,BRAF,MLST8,PIK3CA,HIF1A,PRKAA2,PRKAA1,RPS6KA1, ULK1,EIF4E2,EIF4B,EIF4EBP1,TSC2,CAB39L,RPS6,PIK3CD,STK11,AKT2, VEGFB,PDPK1,MTOR
P53_SIGNALING_PATHWAY	1.88	<0.001	0.011	GTSE1,CCNB1,CDK1,CHEK1,CCNB2,RRM2,CDK4,CASP8,CDK2,ATR, CDKN2A,APAF1,PPM1D,PIDD1,CCNE2,ATM,CCNG2,CASP3,RCHY1,BAX, TP73,COP1,BID,MDM4,PMAIP1,MDM2,IGFBP3,SFN,SIAH1,CCND3, BBC3,PERP,TSC2,TP53,SHISA5,TNFRSF10B,CHEK2,RRM2B
NOTCH_SIGNALING_PATHWA Y	1.87	0.002	0.012	HDAC2,DVL3,DTX2,MAML1,KAT2A,DVL2,ADAM17,NCOR2,RBPJ,HDAC1, NCSTN,CTBP1,NOTCH1,NUMBL,EP300,CREBBP,DTX3,CIR1,APH1A,NOTCH2, SNW1,JAG1,DVL1,MAML3,PSENEN,CTBP2,JAG2,MAML2,PSEN1,DTX3L, DLL3,PSEN2,LFNG,DTX4
REGULATION_OF_AUTOPHA GY	1.86	<0.001	0.012	ATG7,ATG3,BECN1,ATG4B,PIK3C3,ATG5,PRKAA2,ATG12,ATG4C,PRKAA1, ULK1,PIK3R4,BECN2
WNT_SIGNALING_PATHWAY	1.85	<0.001	0.014	TBL1XR1,RUVBL1,MAP3K7,DVL3,RHOA,CSNK2A1,CACYBP,PPP3CB,PLCB1,SMA D2, DVL2,AXIN1,VANGL1,CHD8,PLCB3,GSK3B,MAPK9,CSNK1E,NLK,PPP2CA, RAC1,CTBP1,CSNK2A2,SMAD3,FZD6,PPP2R5D,PRKACB,CAMK2G,CUL1,MAPK8, PRKCA,BTRC,EP300,CREBBP,RBX1,NFATC2,ROCK1,FBXW11,FZD3,ROCK2, PPP2R5E,PPP2R5B,PPP3R1,FZD7,CSNK1A1,PPP3CA,PPARD,PRKX,DVL1,SIAH1, CCND3,SMAD4,LEF1,FRAT2,VANGL2,CTNNB1,NFATC3,NFAT5,SENP2,FZD1, WNT5A,PORCN,TP53,FZD2,PPP2R1A,APC,CTBP2,FZD5,CTNNBIP1,TCF7, CAMK2D,RAC2,PSEN1,PPP2CB,MMP7,AXIN2,SFRP5,DKK1,PLCB2,CSNK2B, TBL1X,PRKCB,MYC,APC2,PRKCG,NFATC1
